# A Good Spray for Nose and Throat Diseases

**Published:** 1898-01

**Authors:** George Brown

**Affiliations:** Atlanta, Ga.


### A GOOD SPRAY FOR NOSE AND THROAT DISEASES

BY GEORGE BROWN, M.D.,
ATLANTA, GA.

The importance of cleanliness in surgical work is everywhere and by everybody recognized as a fundamental principle. It is fully as important in the comparatively restricted limits of nose and throat work as in the broader field of general surgery. I do not think we can urge this point too forcibly or too often.

I believe that in most of the common affections of the nose and throat any remedial treatment directed toward curing the diseased processes in a given case must be preceded by securing, as nearly as possible, a clean condition of the parts. In nasal catarrh (depending upon either atrophied or hypertrophied membranes), in post-nasal catarrh, in ulcerative tonsillitis, the parts must be cleansed of all secretions before we can expect any effect from subsequent applications.

These remarks, the truth of which all will readily admit, are somewhat introductory of what I wish to say about a preparation which I have lately been using for this purpose. I refer to Glycothymoline, manufactured by Kress & Owen, of New York. This is a wine-colored liquid, bland, alkaline and unirritating to the nasal mucous membrane, and when mixed with two or three times its volume of water makes a most reliable spray for the nose and throat. I now use it in the routine treatment of hypertrophic and atrophic rhinitis. In the former condition I use it for its antiseptic and soothing properties after cautery applications to the hypertrophied turbinates. It aids in keeping the nostril clear and in maintaining a healthy condition of the cauterized surface. After its use I have noticed, I think, less inflammatory reaction following the cautery than in other cases. In atrophic rhinitis it is very effectual in removing the crusts and deodorizing the offensive nasal cavities. I have also used it on a cotton tampon after removing spurs from the nasal septum.

I am at present employing two solutions:

| | | | |
|---|---|---|---|
| (1) | ℞ | Glycothymoline | one part. |
| | | Water | two parts. |
| (2) | ℞ | Glycothymoline | one part. |
| | | Dobell's solution | two parts. |

Either of these may be used for the purposes indicated in any of the common hand-ball or compressed air atomizers. Physicians whose offices are supplied with the latter can use these solutions under fifteen or twenty pounds pressure. The Bermingham nasal douche or a good hand atomizer may be prescribed for the patient's own use. I have prescribed it (with equal parts of water) as a gargle in acute tonsillitis and pharyngitis, and my patients have found it pleasant and effective.

I have thought this preparation deserving of the above short notice. No cleansing solution has given me better satisfaction in my nose and throat work. I am now using it daily and with increasing confidence.

